# The Asprosin–OLFR734 module regulates appetitive behaviors

**DOI:** 10.1038/s41421-020-0152-4

**Published:** 2020-04-14

**Authors:** Yang Liu, Aijun Long, Liqun Chen, Liangjie Jia, Yiguo Wang

**Affiliations:** MOE Key Laboratory of Bioinformatics, Tsinghua-Peking Joint Center for Life Sciences, School of Life Sciences, Tsinghua University, 100084 Beijing, China

**Keywords:** Mechanisms of disease, Hormone receptors

Dear Editor,

Organisms need to maintain the balance between energy intake and expenditure for healthy survival. For mammals, eating is the most common process to fuel the body, and ingestive behaviors are well controlled by the neural system in response to peripheral signals, such as nutrients and hormones^1^. In the arcuate nucleus (ARC) of the hypothalamus, Agouti-related peptide-expressing (AgRP) neurons are activated by energy deficit to promote appetitive behaviors^2^. By contrast, proopiomelanocortin (POMC) neurons sense when energy levels are sufficient and inhibit food intake^2^. Hormones, such as neuropeptide Y (NPY), ghrelin, leptin and glucagon-like peptide-1 (GLP-1), and circulating nutrients deliver signals to these neurons^3^. Olfaction also plays an important role in regulating appetitive behavior^1^. The hypothalamus can receive olfactory inputs from olfactory sensory neurons and the olfactory bulb (OB) to coordinate food appreciation and selection^4^.

Asprosin, which is cleaved from fibrillin 1, is a fasting-induced hormone secreted by adipose tissue^5^. Circulating Asprosin binds to the olfactory receptor OLFR734 in the liver to promote hepatic gluconeogenesis via the cAMP-PKA-signaling pathway^5,6^. It is also reported that Asprosin can cross the blood–brain barrier to activate AgRP neurons to stimulate appetite^7^. However, it is still unknown whether OLRF734, as an olfactory receptor and a receptor of Asprosin, mediates appetitive behaviors.

To determine whether OLFR734 regulates appetitive behaviors, we compared the food intake between wildtype (WT) mice and *Olfr734*^−/−^ mice. OLFR734 deficiency significantly decreased the food intake in overnight-fasted mice compared with WT mice (Fig. 1a), especially in the first hour after fasting and at night (dark phase). As a result, the accumulated amount of food intake of *Olfr734*^−/−^ mice is much less than WT mice (Fig. 1b). Under ad lib-feeding conditions, the accumulated food intake is comparable between fed WT and *Olfr734*^−/−^ mice, although *Olfr734*^−/−^ mice ate slightly less at the very beginning of the test than WT mice (Supplementary Fig. S1a, b). In addition, the body weights of WT and *Olfr734*^−/−^ mice are similar under fed or fasted conditions (Supplementary Fig. S1c). Together, these results indicate that OLFR734 promotes fasting-induced food intake in mice.

**Fig. 1:**
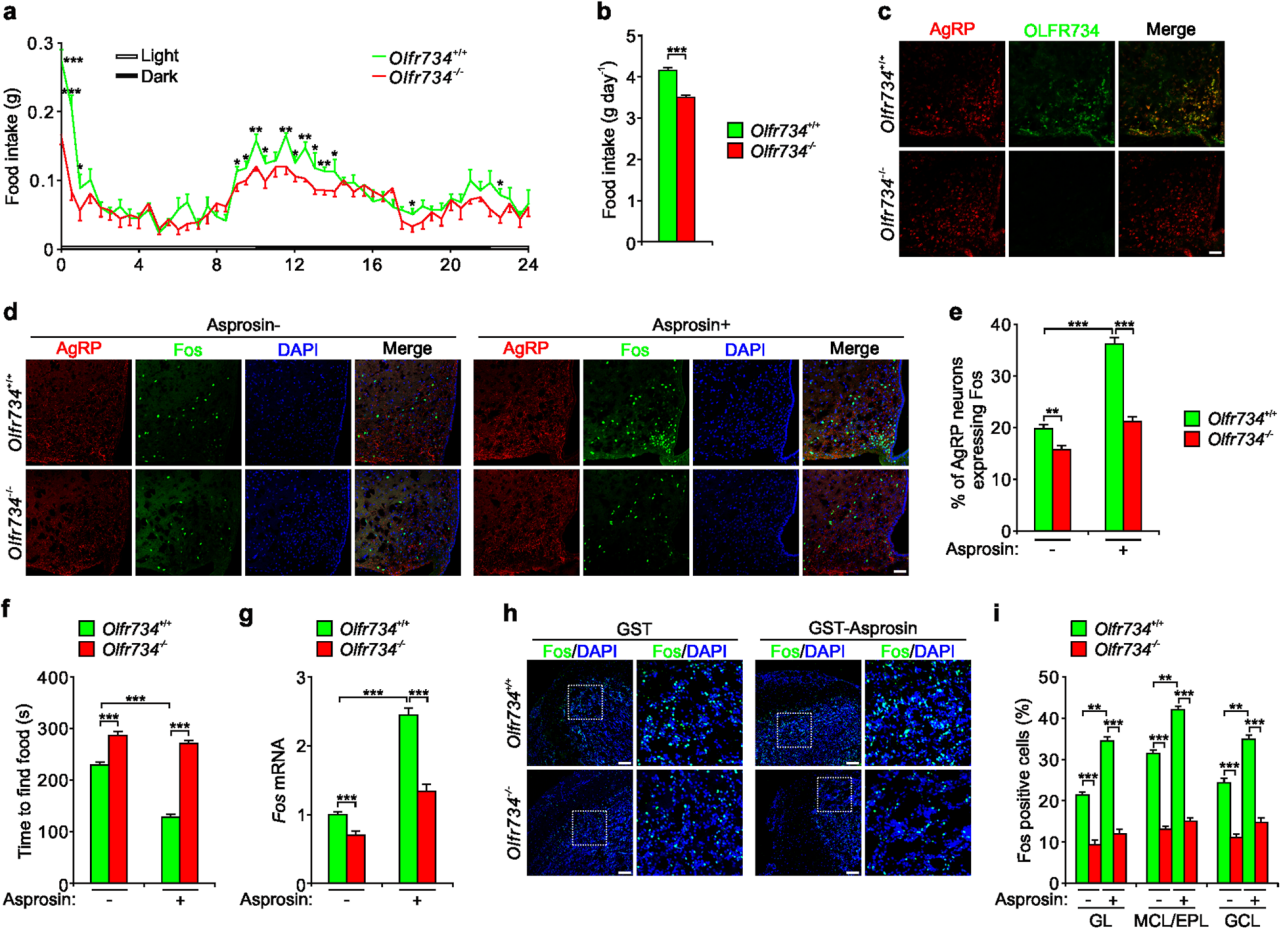
Asprosin improves olfactory performance and activates AgRP neurons via OLFR734. **a**, **b** Food intake curves (**a**) and cumulative food intake (**b**) from 0 to 24 h in WT and *Olfr734*^−/−^ mice immediately after overnight fasting. *n* = 8 mice. **c** Fluorescence in situ hybridization showing the expression of *Olfr734* and *Agrp* in the arcuate nucleus (ARC) of the hypothalamus from WT and *Olfr734*^−/−^ mice. Scale bars, 50 μm. **d**, **e** Fos staining (**d**) and quantitation of Fos-positive cells (**e**) showing neuronal activation of AgRP neurons from WT and *Olfr734*^−/−^ mice administered with GST (Asprosin−) or GST-Asprosin (Asprosin+). Scale bars, 50 μm. *n* = 5 mice. **f** Time taken to find hidden food pellets by WT and *Olfr734*^−/−^ mice administered with GST (Asprosin−) or GST-Asprosin (Asprosin+). *n* = 9 mice. **g** Relative mRNA levels of *Fos* in olfactory bulb extracts from WT and *Olfr734*^−/−^ mice administered with GST (Asprosin−) or GST-Asprosin (Asprosin+). *n* = 5 mice. **h**, **i** Fos staining (**h**) and quantitation of Fos-positive cells (**i**) showing neuronal activation of olfactory bulbs from WT and *Olfr734*^−/−^ mice administered with GST (Asprosin−) or GST-Asprosin (Asprosin+). GL glomerular layer, MCL mitral cell layer, EPL external plexiform layer, GCL granule cell layer. Scale bars, 50 μm. *n* = 8 mice. Data are shown as mean ± sem. *P < 0.05, **P < 0.01, ***P < 0.001.

AgRP neurons in the ARC of the hypothalamus are activated by energy deficit to promote feeding behaviors^2^. To investigate whether OLFR734 can mediate the activation of AgRP neurons, we first identified the expression of *Olfr734* in AgRP neurons. *Olfr734* is expressed in AgRP neurons, as evaluated by fluorescence in situ hybridization (Fig. 1c). The expression of Fos in the AgRP neurons and the proportion of cells positive for Fos (a marker of neuronal activation) are much lower in *Olfr734*^−/−^ mice than in WT mice (Fig. 1d, e). In addition, Asprosin administration enhanced Fos staining in AgRP neurons from WT mice but not *Olfr734*^−/−^ mice (Fig. 1d, e). Together, these results show that OLFR734, as a receptor of Asprosin, promotes AgRP neuronal activity.

Since olfaction also plays an important role in regulating appetitive behavior, we investigated whether the Asprosin–OLFR734 module affects olfactory performance. Olfactory performance is enhanced by fasting and reduced by feeding in rodents and humans^8^. Since Asprosin is enhanced during fasting and OLFR734 is also highly expressed in olfactory epithelium and OB, we used a buried food test^9^ to investigate whether Asprosin affects mouse olfaction via OLFR734. Plasma Asprosin was enhanced to a similar extent in WT and *Olfr734*^−/−^ mice after fasting, while OLFR734 expression was not affected by fasting (Supplementary Fig. S2a, b). In fasted WT mice, the food finding time was about 50% of that in fed WT mice (Supplementary Fig. S2c). The fasting-induced effect on food finding was much weaker in *Olfr734*^−/−^ mice than WT (Supplementary Fig. S2c). Considering the effect of OLFR734 on smell, we tested whether Asprosin has a similar effect by injecting Asprosin into mice. Without food odor stimulus, Asprosin administration to satiated WT or *Olfr734*^−/−^ mice cannot induce any elevated Fos expression in the OB (Supplementary Fig. S2d). With food odor stimulus, Asprosin administration in WT mice, but not in *Olfr734*^−/−^ mice, increased the expression of *Fos* in the OB and the proportion of cells positive for Fos, and decreased the food finding time (Fig. 1f–i). These results indicate that the Asprosin–OLFR734 axis mediates the fasting-induced increase of olfactory performance.

Olfactory sensitivity and discrimination are impaired in obese or diabetic rodents and humans^10^. Compared to regular diet (RD)-fed mice, mice fed a high fat diet (HFD) for 16 weeks had significantly higher levels of plasma Asprosin (Supplementary Fig. S3a), a decreased proportion of cells positive for Fos (Supplementary Fig. S3b, c) and lower expression of *Fos* (Supplementary Fig. S3d) in the OB. The buried food test further revealed that HFD-fed mice took more time to find food pellets than RD-fed mice (Supplementary Fig. S3e). These results indicate that HFD feeding impaired olfactory performance in mice, and the increased plasma Asprosin levels in HFD-fed mice was not sufficient to reverse the deteriorated olfaction. Consistent with this notion, administration of additional Asprosin increased the proportion of Fos-positive cells and the expression of *Fos*, and decreased the food finding time in HFD-fed mice (Supplementary Fig. S3a–e). Together, these results demonstrate that Asprosin can enhance olfactory performance and partially rescue HFD-induced olfactory impairment.

Previous studies showed that OLFR734 in the liver, as a receptor of Asprosin, promotes hepatic gluconeogenesis^6^. Here, we reported that OLFR734 in the nervous system, as a receptor of Asprosin, stimulates appetitive behavior by improving olfactory performance and activating AgRP neurons. The decreased food intake in *Olfr734*^−/−^ mice in the first hour after fasting (Fig. 1a) may reflect the importance of olfaction in food seeking behavior. The OLFR734–Asprosin axis mediates two important fasting-related functions to help organisms to acquire more energy. Thus, it will bell interesting to determine how the two processes coordinate energy homeostasis though crosstalk between peripheral organs and the central nervous system.

Our finding that Asprosin alone cannot activate neurons in the OB (Supplementary Fig. S2d) indicates that Asprosin itself is a potential amplifier, but not an inducer, of odor-stimulated signals. It is possible that Asprosin, as an internal cue, and odorants, as external cues, coordinate smell and food seeking behavior via OLFR734. Therefore, it is important to determine which odorants are agonists of OLFR734. Asprosin and other hormonal factors^10^ can improve HFD-induced olfactory impairment. However, it should be noted that Asprosin is a gluconeogenic hormone and activation of OLFR734 signaling may increase the risk of hyperglycemia. In contrast, immunologic neutralization of Asprosin to relieve insulin resistance and obesity in mice may worsen olfaction under metabolic stress.

## Supplementary information

Supplementary Information

## Supplementary information

**Supplementary Information** accompanies the paper at (10.1038/s41421-020-0152-4).
