# Supplementary material for: The Asprosin–OLFR734 module regulates appetitive behaviors
Source: Cell Discov. 2020 Apr 14;6:19. doi: 10.1038/s41421-020-0152-4 (PMC7154029; doi:10.1038/s41421-020-0152-4)
Supplement: Supplementary file 1 — Supplementary Information [file 41421_2020_152_MOESM1_ESM.pdf]

Materials and methods

Mouse strains

Mice were housed in colony cages with a 12 h light/dark cycle in a temperature-controlled environment with free access to water and diet. *Olfr734*$^{-/-}$ mice have been described previously[1]. For high fat diet challenge, mice were provided with a high fat diet (D12492, Research Diets) for 16 weeks. All mice were maintained on a C57BL/6J background. All animal experiments were approved by the Animal Care and Use Committee at Tsinghua University.

Food finding test

The test was performed as previously described[2]. Briefly, the mice were individually housed for 3 days and habituated to small food pellets before the experiment started. To evaluate the effect of Asprosin on olfaction, mice were fasted overnight and exposed to recombinant GST or GST-Asprosin (1 mg kg^{-1}) via subcutaneous injection 0.5 hr before the food finding test. The mice were given up to 15 min to find a hidden perforated Eppendorf tube containing food pellets under 3 cm of bedding.

Food intake test

Food intake was measured for individually housed mice with an automatic animal feeding system (BiolinkOptics) according to the manufacturer's manual. Precision pellet food (5TUM, TestDiet) was provided to every mouse and each eating event was recorded by the instruments for at least 48 hrs.

Protein expression and purification

Mouse Asprosin was expressed and purified as previously described[1]. Briefly, bacteria expressing GST and GST-Asprosin were centrifuged at 4,000 rpm and resuspended in lysis buffer (50 mM HEPES, pH 7.4, 150 mM NaCl, 1% Triton X-100, protease inhibitor cocktail and 1 mM PMSF). After sonication on ice, the lysate was centrifuged at 15,000 rpm for 20 min and the supernatant was incubated with Glutathione-Agarose (Thermo, 16101), which had been equilibrated in equilibration buffer (10 mM phosphate buffer, pH 7.4, 150 mM NaCl) for 2 hrs at 4 ºC. After sufficient washes with the equilibration buffer, GST and GST-Asprosin were eluted with a buffer containing 10 mM reduced glutathione and 50 mM Tris-HCl, pH 8.0 at 4 ºC. Protein solutions were concentrated with a 10-kDa cut-off Centricon filter unit (EMD Millipore, MRCPRT010) and further purified on a HiTrap Desalting column (GE Healthcare, 17140801) to eliminate LPS and exchange the buffer for a PBS-glycerol buffer (pH 8.0).

RNA extraction and quantitative PCR

Total RNA from cells or mouse tissues was extracted using a Total RNA Purification kit (Omega, R6812-02). cDNA was obtained using the Revert Aid First Strand cDNA Synthesis kit (Thermo, K1622). RNA levels were measured with the LightCycler 480 II (Roche) as previously described[1]. The following primers were used for qPCR:

Actin-Forward: 5'-GTCCACCCCGGGGAAGGTGA-3'

Actin-Reverse: 5'-AGGCCTCAGACCTGGGCCATT-3'

Fos-Forward: 5’-GGGAATGGTGAAGACCGTGT-3'
Fos-Reverse: 5’-CCGTTCCCTTCGGATTCTCC-3'
Olfr734-forward: 5'-CGCCGGCTCTGTTGTATCTTA-3'
Olfr734-reverse: 5'-GGCCCACAAAATGGGAGTCG-3'

Immunostaining

The assays were performed as previously described[1]. Mouse olfactory bulbs and hypothalamus were cut into frozen slices (8 μm), then stained with anti-Fos antibody (ab190289, Abcam) or anti-AgRP antibody (GT15023, Neuromics).

Fluorescence in situ hybridization

Fluorescein-labelled RNA probes were prepared using a FISH Tag™ RNA Multicolor Kit according to the manufacturer’s instructions (Invitrogen). Gene fragments of *Olfr734* and *Agrp* were amplified using the followed primers, and cloned in pSPT19. Two-color fluorescence *in situ* hybridization was performed as described[3]. The fluorescein-labelled probes were visualized with Alexa 488 and Alexa 594. The following primers were used:

Olfr734-forward: 5'- ATGGAACCTGCAAATGAT -3'
Olfr734-reverse: 5'- AATGGGAATCAAGCCTGA -3'
Agrp-forward: 5'- CCATATAAGCTCAGGGCA -3'
Agrp-reverse: 5'- GCGGAGAACGAGACTCGC -3'

Sandwich ELISA

Endogenous Asprosin sandwich ELISA was performed as previously described[1]. Briefly, a rabbit polyclonal anti-FBN1 antibody (Abcam, ab53076) against Asprosin was used as the capture antibody, and a mouse anti-FBN1 monoclonal antibody (Abcam, ab124334) was used as the detection antibody. An anti-mouse secondary antibody linked to HRP and TMB solution were used to generate a signal, and absorbance at 450 nm was detected.

Statistical analyses

Age- and weight-matched male mice were randomly assigned to groups for the experiments. No animals were excluded from statistical analyses, and the investigators were not blinded in the studies. All studies were performed on at least three independent occasions. Results are reported as mean ± s.e.m. Comparison of different groups was carried out using two-tailed unpaired Student’s t-test or two-way ANOVA. Differences were considered statistically significant at $P < 0.05$.

REFERENCES

1 Li, E. *et al.* OLFR734 Mediates Glucose Metabolism as a Receptor of Asprosin. *Cell Metab* **30**, 319-328 e318, doi:10.1016/j.cmet.2019.05.022 (2019).

2 Yang, M. & Crawley, J. N. Simple behavioral assessment of mouse olfaction. *Curr Protoc Neurosci* **Chapter 8**, Unit 8 24, doi:10.1002/0471142301.ns0824s48 (2009).

3 Kosman, D. *et al.* Multiplex detection of RNA expression in Drosophila embryos. *Science* **305**, 846, doi:10.1126/science.1099247 (2004).

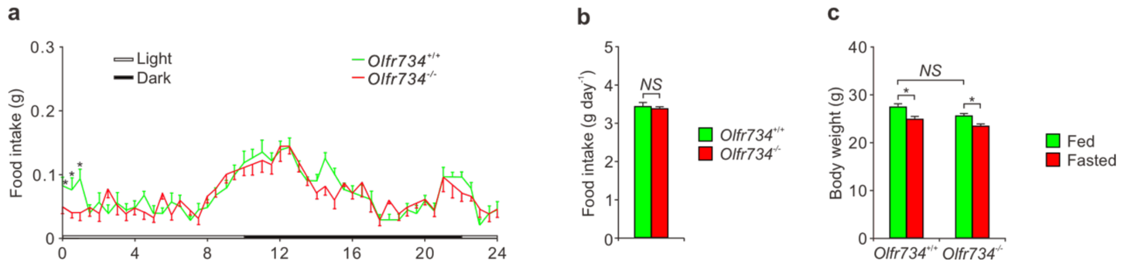

Supplementary Figure S1: Effect of OLFR734 deficiency on food intake and body weight.

a-**b** Food intake curves (**a**) and cumulative food intake (**b**) from 0-24 hrs in fed WT and *Olfr734*$^{-/-}$ mice. n = 8 mice. **c** Body weight of overnight-fasted or *ad lib*-fed WT and *Olfr734*$^{-/-}$ mice. n = 8 mice. *NS*, no statistical significance. Data are shown as mean ± s.e.m. **P* < 0.05.

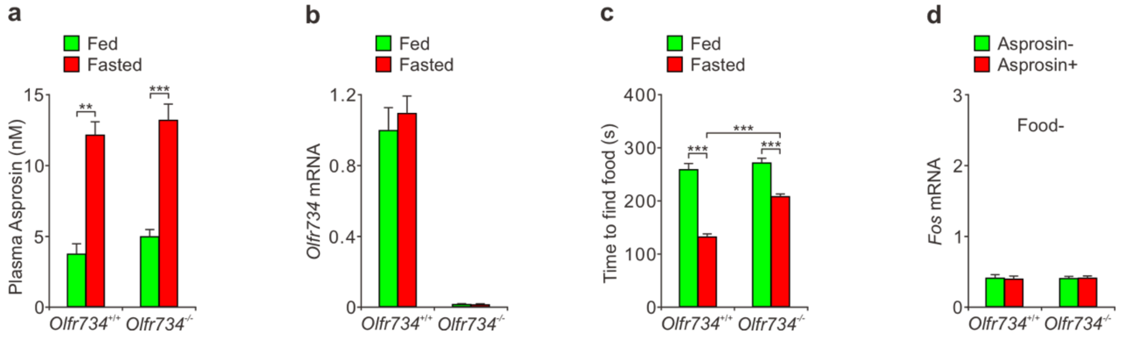

Supplementary Figure S2: The Asprosin-OLFR734 axis modulates olfactory performance.

a Plasma Asprosin levels in WT and *Olfr734*$^{-/-}$ mice fed or fasted overnight. n = 5 mice. **b** Relative levels of *Olfr734* mRNA in olfactory bulb extracts from WT and *Olfr734*$^{-/-}$ mice fed or fasted overnight. n = 5 mice. **c** Time taken to find hidden food pellets by WT and *Olfr734*$^{-/-}$ mice fed or fasted overnight. n = 9 mice. **d** Relative levels of *Fos* mRNA in olfactory bulb extracts from WT and *Olfr734*$^{-/-}$ mice administered with GST (Asprosin-) or GST-Asprosin (Asprosin+) in the absence (Food-) of hidden food pellets. n = 5 mice. Data are shown as mean ± s.e.m. ***P* < 0.01, ****P* < 0.001.

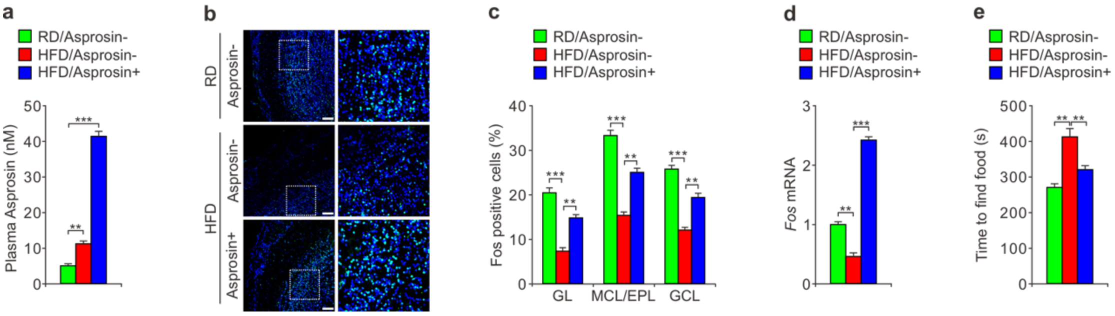

Supplementary Figure S3: Effect of Asprosin on olfactory performance in mice fed on a high fat diet.

a Plasma Asprosin levels of mice administered with GST (Asprosin-) or GST-Asprosin (Asprosin+). **b-c** Fos staining (**b**) and quantitation of Fos-positive cells (**c**) showing neuronal activation of olfactory bulbs from mice fed with a RD or HFD for 16 weeks. GL, glomerular layer; MCL, mitral cell layer; EPL, external plexiform layer; GCL, granule cell layer. Scale bars, 50 μm. **d-e,** Effect of Asprosin on relative *Fos* mRNA levels (**d**) and time taken to find hidden food pellets (**e**) in mice administered with GST (Asprosin-) or GST-Asprosin (Asprosin+). RD, regular diet. HFD, high fat diet. Data are shown as mean ± s.e.m. $^{**}P < 0.01$, $^{***}P < 0.001$. n = 5 mice.
